# Demographics and outcomes of hepatitis B and D: A 10-year retrospective analysis in a Swiss tertiary referral center

**DOI:** 10.1371/journal.pone.0250347

**Published:** 2021-04-27

**Authors:** Joana Vieira Barbosa, Roland Sahli, Vincent Aubert, Aziz Chaouch, Darius Moradpour, Montserrat Fraga

**Affiliations:** 1 Division of Gastroenterology and Hepatology, Lausanne University Hospital and University of Lausanne, Lausanne, Switzerland; 2 Division of Gastroenterology and Hepatology, Beth Israel Deaconess Medical Center, Harvard Medical School, Boston, MA, United States of America; 3 Institute of Microbiology, Lausanne University Hospital and University of Lausanne, Lausanne, Switzerland; 4 Division of Immunology and Allergy, Lausanne University Hospital and University of Lausanne, Lausanne, Switzerland; 5 Division of Biostatistics, Center for Primary Care and Public Health (Unisanté), University of Lausanne, Lausanne, Switzerland; University of Cincinnati College of Medicine, UNITED STATES

## Abstract

**Background:**

Hepatitis B virus (HBV) is a major global health challenge with approximately 250–350 million chronically infected individuals. An improved understanding of the demographic features and outcomes of chronic HBV infection and hepatitis D virus (HDV) infection in low-endemic areas may improve prevention, early identification and management both at individual and community levels. Here, we retrospectively analyzed the demographic and clinical characteristics, treatment rates and outcomes of adult patients with chronic HBV infection with or without HDV coinfection examined at Lausanne University Hospital, Switzerland over a 10-year period.

**Methods:**

We analyzed the medical records of all adult patients with chronic HBV and HDV infection examined in our center between 2007 and 2016. Liver-related outcome was defined as the occurrence of cirrhosis, hepatocellular carcinoma, liver transplantation or liver-related death. Analyses were performed using logistic regression and results were reported as odds ratio (OR) and 95% confidence interval (CI).

**Results:**

Of 672 consecutive patients, 421 (62.6%) were male, median age was 36 years (interquartile range, 28–46 years), and 233 (34.7%) were of African origin. The prevalence of HDV coinfection was 7.1% and the proportion of anti-HDV-positive patients with detectable HDV RNA was 70.0%. In multivariate analysis, HDV coinfection was the strongest predictor for liver-related outcome (OR 6.06, 95% CI 2.93–12.54, p<0.001), followed by HBeAg positivity (OR 2.47, 95% CI 1.30–4.69, p = 0.006), age (OR per 10-year increase 2.03, 95% CI 1.63–2.52, p<0.001) and sex (OR for female 0.39, 95% CI 0.22–0.71, p = 0.002). The predictive accuracy of the multivariate model was high (receiver operator characteristic area under the curve 0.81).

**Conclusion:**

This retrospective study underscores the importance of migration in the epidemiology of chronic hepatitis B in low-endemic areas. HDV coinfection, HBeAg positivity and age predicted liver-related outcomes while female sex had a protective effect.

## Introduction

Hepatitis B virus (HBV) is a major global health challenge with approximately 250–350 million chronically infected individuals. Despite the availability of highly effective vaccines and antiviral therapies, chronic hepatitis B (CHB) is responsible for close to one million deaths per year, mainly due to complications of cirrhosis and hepatocellular carcinoma (HCC) [1–4].

The natural course and clinical outcome of HBV infection are determined by the complex interaction between the virus and the host [5]. Clinical manifestations of chronic HBV infection are broad, ranging from minimal inflammatory activity and fibrosis to active hepatitis with progressive fibrosis, cirrhosis, decompensated liver disease and/or HCC. Sustained HBV replication and liver injury are well-known risk factors for the development of HCC and treatment with nucleos(t)ide analogues does not eliminate the risk of HCC [6]. Thus, new antiviral strategies targeting different steps of the viral life cycle and the host immune response are currently being pursued, with the aim to reach HBV cure [7].

The prevalence of HBV infection varies widely in different geographic areas. HBV infection is highly endemic in sub-Saharan Africa and Asia, with a high-intermediate (5–7.99%) to high prevalence (≥ 8%), with the majority of individuals becoming infected in the perinatal period or early childhood [1–3, 8]. Migration is currently increasing the prevalence of HBV infection in low-endemic areas such as Central and Western Europe [1, 9].

Hepatitis D virus (HDV) is a subviral agent whose propagation depends on the hepatitis B surface antigen (HBsAg). An estimated 12 million people worldwide have serological evidence of HDV infection, with a high prevalence in Mongolia (36.9%), the Republic of Moldova as well as Western and Central Africa (> 10%) [10]. Chronic hepatitis D (CHD) is considered to be the most severe form of chronic viral hepatitis [11].

In Switzerland, the annual notification rate of chronic HBV infection is about 15 cases per 100,000 population [12]. However, the characteristics of CHB and CHD in Switzerland are not well known and, more importantly, contemporary data on treatment rates and patient outcomes in a real-life setting are scarce. Moreover, the epidemiology of HBV in Switzerland is continually evolving due to migration from areas with high prevalence. In this context, a study from Switzerland showed that HBV genotype D was the most prevalent overall, whereas genotype A was the predominant genotype among people born in Switzerland [13].

Here, we analyzed the demographic and clinical characteristics, treatment rates and outcomes of 672 consecutive adult patients with chronic HBV infection with or without HDV coinfection examined in the hepatology outpatient clinic of Lausanne University Hospital over a 10-year period.

## Patients and methods

### Study population and design

This study is a retrospective analysis of medical data from the outpatient clinic of the Division of Gastroenterology and Hepatology of Lausanne University Hospital (Centre Hospitalier Universitaire Vaudois, CHUV), a tertiary referral center serving the south-western region of Switzerland with about 10,000 gastroenterology and hepatology outpatient consultations per year. Patients are referred to our outpatient liver clinic by primary and specialized care providers, regional hospitals or following a hospitalization in our center.

Patients and data for the present study were identified after merging a list of all positive HBsAg test results obtained at the CHUV between January 1, 2007 and December 31, 2016 with a list of all patients evaluated in our outpatient clinic with a diagnosis of chronic HBV infection. After identifying all patients fulfilling our inclusion criteria, we reviewed electronic medical records and medical archives. All data were anonymized prior to analysis. The study was approved by the Ethics Committee for Research of the Canton de Vaud, Switzerland (protocol number 2018–01901). Informed consent for this study was waived by the Ethics Committee for Research of the Canton de Vaud, Switzerland.

Inclusion criteria for the study were: i) all consecutive patients examined at our outpatient clinic between January 2007 and December 2016, ii) > 16 years of age, and iii) documented chronic HBV infection as defined by the presence of a positive HBsAg for more than 6 months or—in the absence of 6 months follow-up—a clinical history compatible with chronic infection [1]. Patients with acute hepatitis B, as defined by the presence of HBsAg for less than 6 months and a clinical course compatible with recent HBV infection, were excluded.

### Baseline evaluation and follow-up

Demographic, clinical, laboratory and histological data were retrieved from electronic medical records and medical archives. Demographic data were assessed at baseline and included age, sex and region of origin. Laboratory parameters were assessed at the first visit and last follow-up exam performed at the time of data collection. These included alanine aminotransferase (ALT), hepatitis B surface and e antigen (HBsAg and HBeAg), HBV DNA, anti-HDV serology and HDV RNA, anti-hepatitis C virus (HCV) serology and HCV RNA.

Liver stiffness was assessed by transient elastography (FibroScan^®^, Echosens, Paris, France) at the time of or within 6 months from the first evaluation. Measurements were considered valid if > 60% were successful and their interquartile range (IQR) was < 30% of the median. Based on previous studies in CHB [14, 15], liver stiffness > 7.2 kPa was used to define significant fibrosis (Metavir stage ≥ 2). Cirrhosis was diagnosed histologically in the majority of patients (64/85) or based on obvious clinical, laboratory and/or imaging features. Necroinflammatory activity and fibrosis were coded as none/minimal (Metavir fibrosis stage F0/F1), moderate (Metavir fibrosis stage F2) and severe or advanced (Metavir fibrosis stage F3/F4).

Liver-related outcome was defined as the occurrence of cirrhosis, HCC, liver transplantation or liver-related death.

### Classification of chronic hepatitis B virus infection

Chronic HBV infection was classified according to the latest European Association for the Study of the Liver (EASL) Clinical Practice Guidelines (CPG) [1]. In line with these, the upper limit of the norm (ULN) for ALT was defined as 40 IU/l for men and women. Hence, patients were assigned to one of the following groups: a) HBeAg-positive chronic infection (positive HBeAg, ALT level ≤ 40 IU/l, HBV DNA > 10^7^ IU/ml on two or more occasions at least 6 months apart, and none or minimal necroinflammatory activity and/or fibrosis); b) HBeAg-positive CHB (positive HBeAg, ALT > 40 IU/L, HBV DNA > 10^4^ IU/ml and moderate or severe/advanced necroinflammatory activity and/or fibrosis); c) HBeAg-negative chronic infection (negative HBeAg, ALT ≤ 40 IU/l and HBV DNA < 2,000 IU/ml on two or more occasions at least 6 months apart, and none or minimal necroinflammatory activity and/or fibrosis); d) HBeAg-negative CHB (negative HBeAg, ALT > 40 IU/l, HBV DNA > 2,000 IU/ml, and moderate or severe/advanced necroinflammatory activity and/or fibrosis); and e) HBeAg-negative indeterminate group (negative HBeAg, ALT ≤ 40 IU/l and HBV DNA < 2,000 IU/ml on one occasion or ALT ≤ 40 IU/l and HBV DNA < 20,000 IU/ml on two or more occasions at least 6 months apart, and none or minimal necroinflammatory activity and/or fibrosis). Of note, patients assigned to the indeterminate group belonged most likely to the group of patients with HBeAg-negative chronic infection. However, assignment to the latter group could not be made with certainty because of i) the absence of follow-up for more than 6 months after initial evaluation; ii) the absence of ALT and/or HBV DNA determination at follow-up; or iii) an HBV DNA between 2,000 IU/ml and 20,000 IU/ml (in the presence of a normal ALT and none or minimal fibrosis). In the absence of liver biopsy, liver stiffness > 7.2 kPa was used to define moderate to severe fibrosis.

### Statistical analyses

Continuous variables were summarized as median (IQR) and categorial variables as frequency (percentage). Baseline demographic and clinical variables were compared using the χ^2^ test or the Fisher’s exact test for categorical variables and the Mann-Whitney or Kruskal-Wallis test for continuous variables. Univariate and multivariate analyses were performed using logistic regression and results were reported as odds ratio (OR) and 95% confidence interval (95% CI). After screening all baseline variables, covariates for the multivariable model were retained based on clinical relevance and/or association with liver-related outcome at p < 0.05 in univariate regression models. The final model was adjusted for age, sex, region of origin, HBeAg status and HDV coinfection. The predictive performance of the model for liver-related outcome was further assessed by calculating the area under the curve (AUC) of the receiver-operating curve. All statistical analyses were performed using Stata 14.0 (Stata Corp, College Station, TX, USA). A two-tailed p value < 0.05 was considered significant.

## Results

### Baseline characteristics of the cohort

Systematic chart review identified 672 patients who met the inclusion criteria. Table 1 summarizes demographic, clinical, laboratory and histological characteristics of the patients. Four hundred and twenty-one (62.6%) were male; median age was 36 years (IQR, 28–46 years). All patients had an HBeAg test available. One hundred two patients (15.2%) had positive HBeAg and the majority of these had CHB (S1 Fig). Five hundred seventy patients (84.8%) had negative HBeAg. HBeAg-positive chronic infection ("immunotolerant state") was present in 12 (1.8%) patients while HBeAg-negative chronic infection ("inactive HBsAg carrier state") was present in 146 (21.7%) or 302 (44.9%) if the HBeAg-negative indeterminate group is added to this group. Patients with HBeAg-positive chronic infection were younger than those with HBeAg-positive CHB (median age 23 *vs*. 34 years, p = 0.007). Among the 358 patients with CHB, 268 (74.9%) had negative HBeAg.

**Table 1:** Characteristics of patients included in the study.

	HBeAg-positive		HBeAg-negative			Total (n = 672)	p value
	Chronic infection (n = 12)	Chronic hepatitis (n = 90)	Chronic infection (n = 146)	Indeterminate (n = 156)	Chronic hepatitis (n = 268)		
**Age at enrolment** (years), median (IQR)	23 (13)	34 (17)	37 (17)	35 (16)	39 (19)	36 (18)	< 0.001
**Patients by age group (%)**							0.005
< 30 years	9 (75.0)	34 (37.8)	43 (29.5)	46 (29.5)	66 (24.6)	198 (29.5)	
30 to < 40 years	2 (16.7)	27 (30.0)	47 (32.2)	56 (35.9)	74 (27.6)	206 (30.7)	
40 to < 50 years	0 (0)	19 (21.1)	35 (24.0)	32 (20.5)	68 (25.4)	154 (22.9)	
≥ 50 years	1 (8.3)	10 (11.1)	21 (14.4)	22 (14.1)	60 (22.4)	114 (17.0)	
**Male sex** (%)	4 (33.3)	67 (74.4)	75 (51.4)	80 (51.3)	195 (72.8)	421 (62.6)	< 0.001
**Female sex** (%)	8 (66.7)	23 (25.6)	71 (48.6)	76 (48.7)	73 (27.2)	251 (37.4)	
**Region of origin** (%)							0.002
Africa	4 (33.3)	22 (24.4)	75 (51.4)	61 (39.1)	71 (26.5)	233 (34.7)	
Central and Western Europe	3 (25.0)	26 (28.9)	29 (19.9)	37 (23.7)	79 (29.5)	174 (25.9)	
Eastern Europe	1 (8.3)	16 (17.8)	25 (17.1)	34 (21.8)	61 (22.8)	137 (20.4)	
Asia	4 (33.3)	26 (28.9)	17 (11.6)	23 (14.7)	53 (19.8)	123 (18.3)	
Other^1^	0 (0)	0 (0)	0 (0)	1 (0.6)	4 (1.5)	5 (0.8)	
**Coinfections** (%)							
Positive anti-HDV^2^	1 (8.3)	5 (5.8)	6 (4.2)	2 (1.4)	32 (12.1)	46 (7.1)	0.001
Positive HDV RNA^2^	0 (0)	1 (1.1)	1 (0.7)	2 (1.3)	24 (9.0)	28 (4.2)	0.017
HCV	0 (0)	1 (1.1)	0 (0)	1 (0.6)	16 (6.0)	18 (2.7)	0.008
**ALT baseline** (IU/l), median (IQR)	29 (10.5)	71 (70)	25 (14)	23 (12.5)	49 (47.5)	34 (33.5)	< 0.001
**ALT last f/u** (IU/l), median (IQR)	28 (14)	39 (29)	23 (10)	24 (10)	34 (26.5)	28 (19)	< 0.001
**HBV DNA baseline** (log_10_ IU/mL), median (IQR)	8.2 (0.2)	7.1 (3.4)	2.2 (2.7)	3.0 (1.6)	3.0 (3.1)	2.9 (2.5)	< 0.001
**HBV DNA last f/u** (log_10_ IU/mL), median (IQR)	8.2 (2.1)	0 (2.5)	2.0 (2.7)	3.4 (1.0)	0 (2.0)	1.6 (2.9)	< 0.001
**Liver stiffness** (kPa), median (IQR)	4.6 (1.7)	6.6 (4.6)	4.8 (1.8)	4.3 (1.5)	6.1 (4)	5.2 (2.6)	< 0.001
**Patients with a liver biopsy** (%)	4 (33.3)	63 (70.0)	22 (15.1)	18 (11.5)	174 (64.9)	281 (41.8)	< 0.001
Necroinflammatory activity							0.036
None or discrete	4 (33.3)	32 (35.6)	21 (14.4)	17 (10.9)	105 (39.2)	179 (26.6)	
Moderate	0	18 (20.0)	0	0	38 (17.2)	56 (8.3)	
Severe	0	6 (6.7)	0	0	16 (6.0)	22 (3.3)	
Fibrosis							< 0.001
None or discrete (Metavir F0/F1)	4 (33.3)	22 (24.4)	22 (15.1)	18 (11.5)	72 (26.9)	138 (20.5)	
Moderate (Metavir F2)	0	15 (16.7)	0	0	31 (15.6)	46 (6.8)	
Advanced (Metavir F3/F4)	0	27 (30.0)	0	0	70 (26.1)	97 (14.4)	
**Patients with f/u exam** (%)	10 (83.3)	73 (81.1)	146 (100)	66 (42.3)	221 (82.5)	516 (76.8)	< 0.001
**Antiviral treatment before baseline** (%)	2 (16.7)	20 (22.2)	1 (0.7)	4 (2.6)	83 (31.0)	110 (16.4)	< 0.001
**Antiviral treatment at last f/u** (%)	1 (8.3)	64 (71.1)	5 (3.4)	2 (1.3)	146 (54.5)	218 (32.4)	< 0.001
**Patients with liver-related outcome** (%)	0 (0)	21 (23.3)	0 (0)	0 (0)	69 (25.8)	90 (14.0)	< 0.001
Cirrhosis (%)	0 (0)	20 (22.2)	0 (0)	0 (0)	65 (24.7)	85 (12.7)	< 0.001
Hepatocellular carcinoma (%)	0 (0)	5 (5.6)	0 (0)	0 (0)	22 (8.2)	27 (4.0)	< 0.001
Liver transplantation (%)	0 (0)	1 (1.1)	0 (0)	0 (0)	6 (2.2)	7 (1.0	0.134
Liver-related death (%)	0 (0)	2 (2.2)	0 (0)	0 (0)	9 (3.4)	11 (1.6)	0.025
Death all causes (%)	0 (0)	3 (3.3)	1 (0.7)	1 (0.6)	12 (4.5)	17 (2.5)	0.066

^1^This group comprises patients from America and Australia.

^2^Percentages calculated according to the number of anti-HDV tests (N = 648).

The indeterminate group includes HBeAg-negative patients i) without a follow-up appointment; ii) without ALT and HBV DNA determination at follow-up, and iii) with an HBV DNA between 2000 and 20,000 IU/ml, normal ALT and none or minimal fibrosis.

Statistical analyses for continuous variables were made using the Kruskal-Wallis test and χ^2^ test or the Fisher’s exact test were used for categorical variables. ALT, alanine aminotransferase; f/u, follow-up; HBV, hepatitis B virus; HCV, hepatitis C virus; HDV, hepatitis D virus; IQR, interquartile range.

Overall, Africa was the most common region of origin (34.7%), followed by Central and Western Europe (25.9%), Eastern Europe (20.4%) and Asia (18.3%) (Table 1 and S2 Fig).

### Baseline characteristics of HDV-coinfected patients

Characteristics of HDV-coinfected patients are summarized in Table 2. The HDV-positive group was defined by positive anti-HDV serology independently of the presence or absence of detectable HDV RNA.

**Table 2:** Characteristics of patients by HDV status.

	HDV-positive (n = 46)	HDV-negative (n = 602)	p value
**Age at enrolment** (years), median (IQR)	36 (15)	36 (18)	0.915
**Patients by age group** (%)			0.883
< 30 years	13 (28.3)	180 (29.9)	
30 to < 40 years	15 (32.6)	187 (31.1)	
40 to < 50 years	12 (26.1)	135 (22.4)	
≥ 50 years	6 (13.0)	100 (16.6)	
**Male** (%)	29 (63.0)	376 (62.5)	0.937
**Region of origin** (%)			0.194
Africa	23 (50.0)	206 (34.2)	
Central and Western Europe	11 (24.0)	151 (25.1)	
Eastern Europe	8 (17.4)	127 (21.1)	
Asia	4 (8.7)	115 (19.1)	
Other^1^	0 (0)	3 (0.5)	
**Patients with positive HDV RNA** (%)	28 (60.9)	-	-
**ALT baseline** (IU/l), median (IQR)	63 (78.0)	33 (29.0)	< 0.001
**ALT last f/u** (IU/l), median (IQR)	37 (46.0)	28 (18.0)	0.017
**HBV DNA baseline** (log_10_ IU/mL), median (IQR)	1.5 (2.7)	3.0 (2.4)	< 0.001
**HDV RNA baseline** (log_10_ copies/mL), median (IQR)	6.4 (3.0)	-	-
**Liver stiffness** (kPa), median (IQR)	6.5 (3.4)	5.0 (2.4)	< 0.001
**Patients with a liver biopsy** (%)	31 (67.4)	239 (39.7)	< 0.001
Necroinflammatory activity			< 0.001
None or discrete	11 (23.9)	161 (26.7)	
Moderate	9 (19.6)	45 (7.5)	
Severe	8 (17.4)	13 (2.2)	
Fibrosis			0.029
None or discrete (Metavir F0/F1)	8 (17.4)	124 (20.6)	
Moderate (Metavir F2)	5 (10.9)	40 (6.6)	
Advanced (Metavir F3/F4)	18 (39.1)	76 (12.6)	
**Patients with f/u exam** (%)	38 (82.6)	467 (77.6)	0.427
**Patients with liver-related outcome** (%)	17 (37.0)	70 (11.6)	< 0.001
Cirrhosis (%)	17 (37.0)	65 (10.8)	< 0.001
Hepatocellular carcinoma (%)	4 (8.7)	23 (3.8)	0.111
Liver transplantation (%)	1 (2.2)	6 (1.0)	0.404
Liver-related death (%)	3 (6.5)	8 (1.3)	0.009
Death all causes (%)	3 (6.5)	14 (2.3)	0.086

^1^This group comprises patients from America and Australia.

Statistical analyses for continuous variables were made using the Mann-Whitney test and χ^2^ test or Fisher’s exact test were used for categorical variables. f/u, follow-up; HBV, hepatitis B virus; HDV, hepatitis D virus; IQR, interquartile range.

Out of 672 patients, 648 (96.4%) had a test for anti-HDV. Of these, 46 (7.1%) presented a positive serology. Forty anti-HDV-positive patients (87.0%) had a test for HDV RNA by PCR. Of these, 28 had detectable HDV RNA (4.3% of the entire cohort, 70.0% of the tested anti-HDV-positive patients, 60.9% of all anti-HDV-positive patients).

HDV-positive patients had a median age of 36 years (IQR, 29–44 years), with 63.0% being male and 50.0% of African origin. Age, sex and region of origin were similar among HDV-positive *vs*. HDV-negative patients.

Median ALT was significantly higher in HDV-positive *vs*. HDV-negative patients (63 IU/l [IQR, 33–111 IU/l] *vs*. 33 IU/L [IQR, 22–51 IU/l], p < 0.001).

As proposed previously [16], HDV dominance was defined as HDV RNA (log_10_ cp/ml) > HBV DNA (log_10_ IU/ml) and HBV dominance *vice versa*. The majority of patients (92.9%) presented HDV dominance while HBV dominance was found only in two patients (7.1%) (S3 Fig).

### Association between liver stiffness and fibrosis stage

Liver stiffness measurements were available in 474 (78.7%) patients with HBV infection alone and 32 (69.6%) patients with HDV coinfection. Liver biopsy was performed in 239 (39.7%) and 31 (67.4%) of mono- and coinfected patients, respectively. Transient elastography was performed within < 3 months from liver biopsy in all patients. Liver stiffness was significantly higher in HDV-positive as compared to HDV-negative patients (6.5 kPa [IQR, 5.4–8.8 kPa] *vs*. 5.0 kPa [IQR, 4.0–6.4 kPa], p < 0.001).

**Fig 1:**
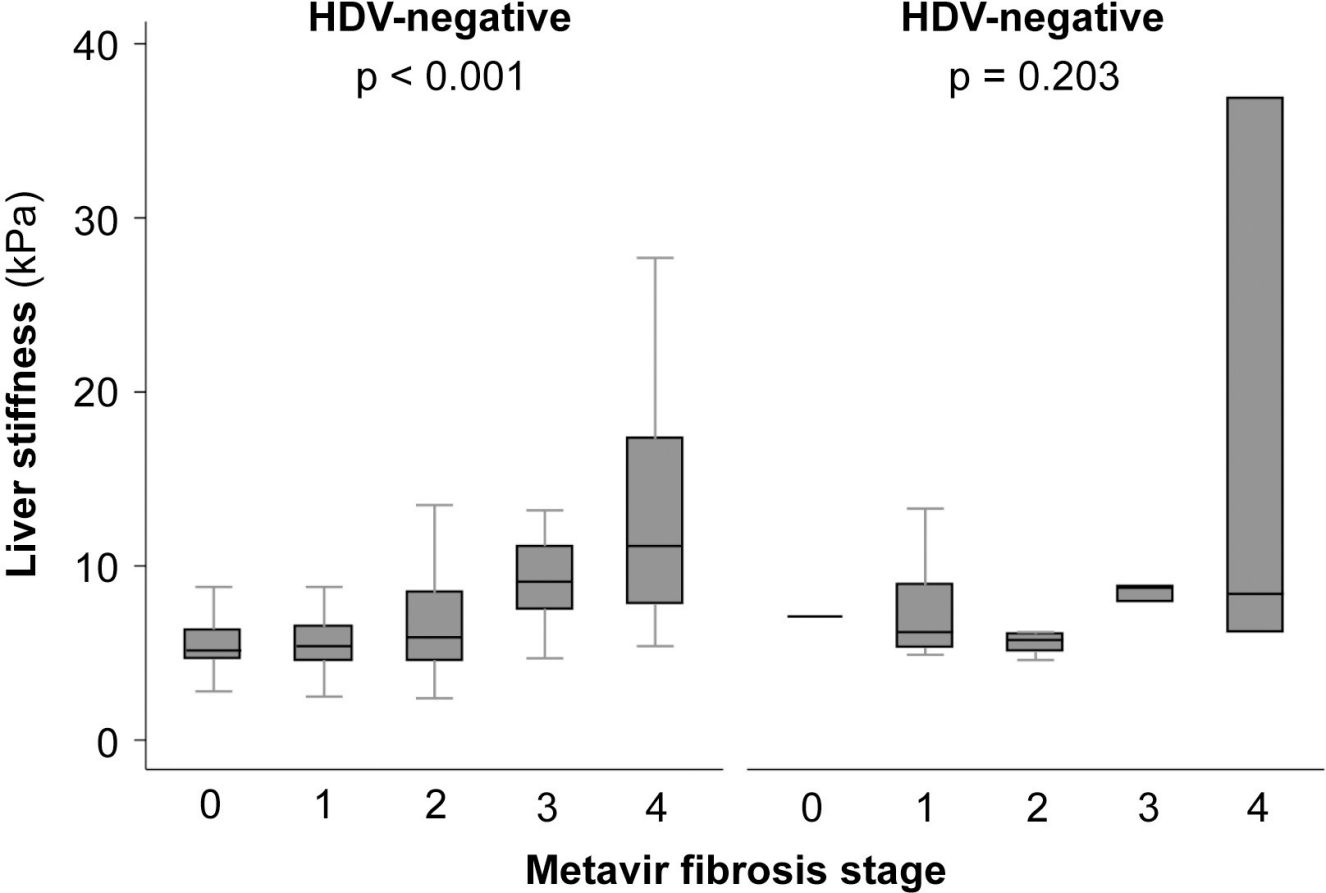
Correlation between liver stiffness as evaluated by transient elastography and histological fibrosis stage according to the absence or presence of hepatitis D virus (HDV) coinfection. The horizontal line within each box represents the median, the limits of each box the interquartile range, and the whiskers the maximum and minimum values. Statistical analyses were performed using Kruskal-Wallis test and p values calculated comparing liver stiffness and Metavir fibrosis stage.

In patients without HDV infection, liver stiffness and histological fibrosis stage were significantly correlated (p < 0.001). However, the correlation was poor in HDV-positive patients (p = 0.203) (Fig 1).

### Treatment eligibility

The EASL CPG published in 2017 recommend antiviral treatment for 1) all patients with HBeAg-positive or -negative CHB; 2) patients with cirrhosis and detectable HBV DNA; 3) patients with HBV DNA > 20,000 IU/ml and ALT > 2 x ULN; and 4) patients with HBeAg-positive chronic infection older than 30 years [1]. Table 3 compares the rates of antiviral treatment in our cohort to treatment indications as defined in the EASL CPG. Among 227 (33.8%) patients eligible for antiviral treatment according to the EASL CPG, 181 (79.7%) were effectively treated, while 46 (20.3%) were not treated. Another 37 (17.0%) patients were treated without fulfilling EASL CPG eligibility criteria for antiviral treatment.

**Table 3:** Comparison between the number of patients eligible for antiviral treatment according to the latest European Association for the Study of the Liver (EASL) Clinical Practice Guidelines (CPG) [1] and patients who were effectively treated.

**Total of patients included in study**, n (%)	672 (100)
**Total of patients eligible for antiviral treatment according to EASL CPG**, n (%)	227 (33.8)
HBV DNA > 2,000 IU/ml, ALT > ULN, at least moderate liver necroinflammation or fibrosis, n	65
Cirrhosis with detectable HBV DNA, n	85
HBV DNA > 20,000 IU/ml and ALT > 2 x ULN, n	34
HBeAg-positive and > 30 years, n	43
**Total of patients under antiviral treatment**, n (%)	218 (32.4)
Patients fulfilling EASL eligibility criteria, n	181
Patients not fulfilling EASL eligibility criteria, n	37
**Patients who fulfill EASL eligibility criteria but were not treated**, n (%)	46 (20.3)
HBV DNA > 2,000 IU/ml, ALT > ULN, at least moderate liver necroinflammation or fibrosis, n	13
Cirrhosis with detectable HBV DNA, n	14
HBV DNA > 20,000 IU/ml and ALT > 2 x ULN, n	6
HBeAg-positive and > 30 years, n	13

In the absence of a liver biopsy, moderate fibrosis was considered when liver stiffness was > 7.2 kPa. HBV, hepatitis B virus; HDV, hepatitis D virus

### Univariate and multivariate analyses of factors associated with liver-related outcomes

Cirrhosis, HCC, liver transplantation or liver-related death occurred in 90 patients: 85 (12.7%) cases of cirrhosis, 27 (4.0%) cases of HCC, 7 (1.0%) liver transplantations and 11 (1.6%) liver-related deaths.

In univariate analysis, factors significantly associated with liver-related outcome included female sex (OR 0.35, 95% CI 0.20–0.60, p < 0.001), age ≥ 40 years (40 to < 50 years: OR 3.45, 95% CI 1.65–7.24, p = 0.001; ≥ 50 years, OR 8.50, 95% CI 4.13–17.50, p < 0.001), origin from Central or Western Europe (OR 2.27, 95% CI 1.30–3.97, p = 0.004), HDV coinfection (OR 4.46, 95% CI 2.33–8.52, p < 0.001) and HBeAg positivity (OR 1.88, 95% CI 1.09–3.24, p = 0.022). Age ≥ 50 years and HDV coinfection were the strongest predictors of the liver-related outcome in the univariate analysis. The event rate in the HDV-positive group was 17/46 (37.0%) *vs*. 70/602 (11.6%) in the HDV-negative group (p < 0.001) (S4 Fig).

**Fig 2:**
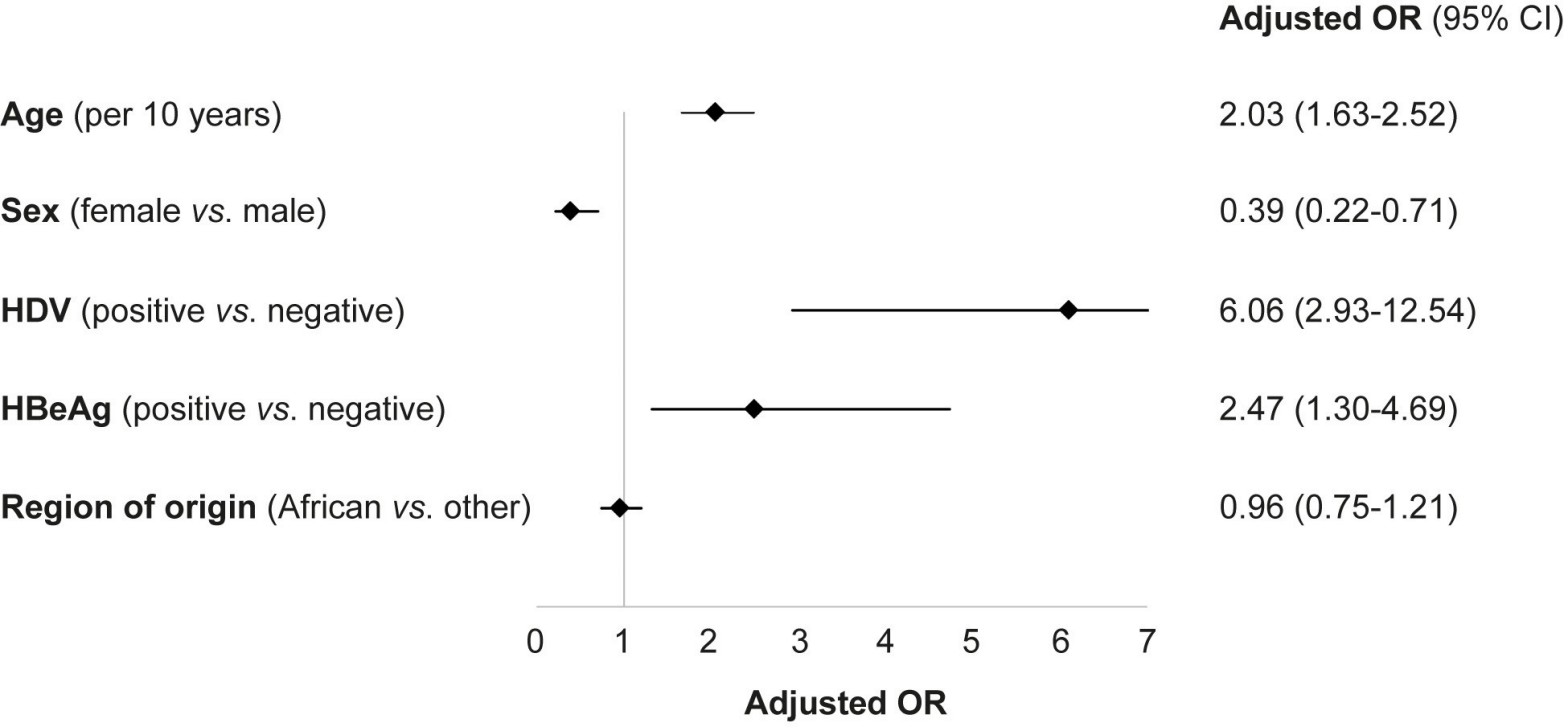
Multivariate analysis for liver-related outcome (cirrhosis, hepatocellular carcinoma, liver transplantation or liver-related death). Analysis was performed by logistic regression, adjusting for all variables in the figure. Results are expressed as adjusted odds ratio (OR) and 95% confidence interval (CI).

Multivariate analysis was adjusted for age, sex, region of origin, HDV coinfection and HBeAg status (Fig 2 and S1 Table). In this analysis, HDV infection was the strongest predictor of liver-related outcome, with HDV-positive patients presenting an OR of 6.06 (95% CI 2.93–12.54, p < 0.001) as compared to HDV-negative patients. Positive HBeAg was also strongly associated with liver-related outcome (OR 2.47, 95% CI 1.30–4.69, p = 0.006), as was age (OR per 10-year increase 2.03, 95% CI 1.63–2.52, p < 0.001) and female sex (OR 0.39, 95% CI 0.22–0.71, p = 0.002).

The prognostic accuracy of the multivariate model for liver-related outcome was high, with an AUC of 0.81 (S5 Fig).

## Discussion

This retrospective study performed in the practice setting of a Swiss tertiary referral center reveals several important findings: i) patients were younger than previously reported in similar studies and mostly of African origin; ii) the prevalence of anti-HDV positivity was 7.1% and over two thirds of the anti-HDV-positive patients tested had detectable HDV RNA; iii) transient elastography lacked accuracy in evaluating fibrosis stage in patients with HDV coinfection; iv) four of five patients eligible for antiviral treatment according to the latest EASL CPG were treated; and v) age, HDV coinfection and HBeAg positivity were strongly associated with cirrhosis, HCC, liver transplantation or liver-related death while female sex had a protective effect.

In our study, median age was 36 years and the male-to-female ratio was 3:2. This is in line with two previous studies from Switzerland [13, 17]. However, our findings are different from those reported in the Hepatitis B Research Network [18], where patients were older (median age 42 years) and without gender predominance in the overall population. One third of our patient population was of African origin, confirming that population movements and migration from highly endemic regions to low-prevalence countries are changing the demographics of CHB, particularly in Central Europe, and represents a public health challenge for host countries [9, 12]. In Europe, the prevalence of chronic HBV infection is estimated to be 1.6% [2], a figure that may be underestimated due to the high number of migrant populations arriving to European Union countries in the last years [9].

Second, 7.1% of our patients were anti-HDV-positive and the majority of them had detectable active viral replication, showing that HDV coinfection remains prevalent in Europe. For the purpose of our study, the HDV-positive group was defined by positive anti-HDV serology independently of the presence or absence of detectable HDV RNA. This choice was made due to the assumption that some coinfected patients may have HDV RNA suppression by HBV [14]. HDV demographics are still not well known and possibly underestimated. In Europe, most studies reported an anti-HDV prevalence of 4–10% among patients with HBV infection [19–21] but migration of individuals from high-to-intermediate-prevalence areas to low-prevalence countries, such as Switzerland, is changing HDV epidemiology [22]. According to the World Health Organization, approximately 12 million individuals are coinfected with HDV worldwide [2, 10]. However, a recent meta-analysis [23] pointed to a much higher prevalence. Thus, large-scale studies are needed in order to better understand the epidemiology, clinical course and outcome of patients with CHD in Europe.

Third, transient elastography did not accurately assess liver fibrosis in patients with HDV infection in our cohort. However, these results need to be interpreted with caution due to the relatively small sample size. Despite being extensively studied and validated in CHB, data on the accuracy of FibroScan^®^ in patients with HDV coinfection is still limited. Hence, prospective larger-scale studies are warranted to confirm these results.

Fourth, antiviral treatment has been demonstrated to prevent cirrhosis, liver failure and HCC [6]. One third of the patients in our cohort were under antiviral treatment at the end of follow-up. When applying the latest EASL CPG of 2017, 79.7% of the patients that had an indication to treatment were treated in our practice setting. Lack of antiviral therapy in one fifth of our patients was primarily due to poor adherence to treatment and follow-up. Moreover, at the moment of data collection, the latest EASL CPG had just been released and most patients had been offered antiviral treatment based on older recommendations. In fact, only 169 (25.1%) (instead of 227 [33.8%]) of our patients have been eligible for antiviral treatment according to the EASL CPG from 2012. The rate of 20.3%, however, is lower than the rates reported in the literature. A recent study [24] from the Polaris Observatory Collaborators, which developed a model for 120 countries using data from a literature review and interviews with experts, estimates that only 5% of the eligible patients for treatment actually received antiviral therapy.

Finally, we show that HDV coinfection, HBeAg, age and female sex are strongly associated with liver-related outcome. In fact, HDV coinfection was the strongest predictor, associated with a 6-fold higher risk of cirrhosis, HCC, liver transplantation or liver-related death. The associations between HDV coinfection and the risk of cirrhosis and HCC [25], HBeAg and the risk of HCC [26] and cirrhosis [27], as well as sex and the risk of HCC [28] have been described. These results underscore the importance of HDV screening in the management of patients with HBV infection [11]. In our cohort, HDV screening has been performed in almost all patients; only 3.6% did not have an anti-HDV test. By comparison, in a prospective cohort of 4673 patients with CHB, the anti-HDV testing rates ranged from 57% before 2003 to 35% thereafter [21]. The lack of HDV screening and the fact that serological tests were less accurate in the past may explain why the epidemiology of CHD is still not fully understood and the real global prevalence remains unknown [29].

Our study presents some limitations. First, the relatively small sample size of patients with HDV coinfection limited our analysis. Secondly, regarding HDV *vs*. HBV viral dominance, our observations were based only on a one-time assessment, therefore not accounting for fluctuations in viral loads over time. Moreover, missing data and loss to follow-up are inherent limitations to a retrospective study design. To our knowledge, however, this is the largest study evaluating the presentation and outcome of hepatitis B and D in Switzerland to date.

In conclusion, our study has potential implications for public health policy and practice, underlining the effect of migration in the demographics of HBV and HDV infection. In spite of the low prevalence of chronic HBV infection in Switzerland and Central Europe, the development of population-wide policies for HBV testing should enable early diagnosis as well as linkage to specialized evaluation and care, particularly in individuals with a personal or family history of migration from an intermediate- to high-prevalence area. The early introduction of antiviral treatment, when indicated, is vital to reduce the rate of adverse liver-related outcomes. This illustrates the urgent need of public health policies to decrease the burden of chronic hepatitis B, even in low endemicity settings. Appropriate HCC surveillance and early implementation of antiviral treatment should be offered to patients with CHB, when indicated, in order to decrease the incidence of new infections and the development of advanced liver disease. Vaccination also stands as an important measure to limit disease and should be actively implemented worldwide. Finally, HDV coinfection was found to be a strong predictor of unfavorable outcome. These patients carry a higher risk for cirrhosis and HCC, which highlights the need for close monitoring and HCC surveillance. In our cohort, transient elastography did not accurately assess liver fibrosis in patients with HDV coinfection. Hence, liver biopsy remains the current gold standard in the evaluation of these patients.

## Supporting information

S1 Table Univariate et multivariate analysis of patient characteristics associated with liver-related outcome (cirrhosis, hepatocellular carcinoma, liver transplantation, liver-related death). Statistical analyses were performed using logistic regression and results were reported as odds ratio (OR) and 95% confidence interval (95% CI). HBV, hepatitis B virus; HDV, hepatitis D virus.
(DOCX)

S1 Fig Percentages of patients assigned to the different categories of chronic hepatitis B virus infection. (TIF)

S2 Fig Regions of origin of the patients included in the study. "Other" comprises patients from America and Australia. Results are expressed in percentages. HBV, hepatitis B virus.
(TIF)

S3 Fig Viral dominance pattern in patients coinfected with hepatitis B virus (HBV) and hepatitis D virus (HDV). (TIF)

S4 Fig Percentages of patients presenting liver-related outcome according to hepatitis D virus (HDV) coinfection status. HCC, hepatocellular carcinoma.
(TIF)

S5 Fig Diagnostic accuracy of the model of multivariate analysis for the prediction of cirrhosis, hepatocellular carcinoma, liver transplantation and liver-related death. The area under the receiver operating characteristic curve for the multivariate model is 0.81.
(TIF)

## Abbreviations

HBV: hepatitis B virus
CHB: chronic hepatitis B
HCC: hepatocellular carcinoma
HDV: hepatitis D virus
HBsAg: hepatitis B virus surface antigen
CHD: chronic hepatitis D
ALT: alanine aminotransferase
HBeAg: hepatitis B e antigen
HCV: hepatitis C virus
IQR: interquartile range
EASL: European Association for the Study of the Liver
CPG: Clinical Practice Guidelines
ULN: upper limit of the norm
OR: odds ratio
AUC: area under the curve
