# Supplementary material for: Demographics and outcomes of hepatitis B and D: A 10-year retrospective analysis in a Swiss tertiary referral center
Source: PLoS One. 2021 Apr 27;16(4):e0250347. doi: 10.1371/journal.pone.0250347 (PMC8078781; doi:10.1371/journal.pone.0250347)
Supplement: S1 Table — Statistical analyses were performed using logistic regression and results were reported as odds ratio (OR) and 95% confidence interval (95% CI). HBV, hepatitis B virus; HDV, hepatitis D virus. (DOCX) [file pone.0250347.s001.docx]

**S1 Table**. **Univariate et multivariate analysis of patient characteristics associated with liver-related outcomes (cirrhosis, hepatocellular carcinoma, liver transplantation, liver-related death).**

|  | **Univariate analysis** | | **Multivariate analysis** | |
| --- | --- | --- | --- | --- |
|  | OR (95% CI) | p value | OR (95% CI) | p value |
| **Age** (per 10 years) | 1.87 (1.56-2.24) | < 0.001 | 2.03 (1.63-2.52) | < 0.001 |
| **Sex** |  |  |  |  |
| Male | 1 (reference) |  | 1 (reference) |  |
| Female | 0.35 (0.20-0.60) | < 0.001 | 0.39 (0.22-0.71) | 0.002 |
| **Region of origin** |  |  |  |  |
| Africa | 1 (reference) |  | 1 (reference) |  |
| Central and Western Europe | 2.27 (1.30-3.97) | 0.004 | 0.99 (0.50-1.97) | 0.988 |
| Eastern Europe | 1.07 (0.54-2.12) | 0.845 | 0.92 (0.44-1.95) | 0.833 |
| Asia | 1.21 (0.61-2.40) | 0.587 | 0.92 (0.42-1.99) | 0.823 |
| **HBeAg-positive** | 1.88 (1.09-3.24) | 0.022 | 2.47 (1.30-4.69) | 0.006 |
| **HDV-positive** | 4.46 (2.33-8.52) | < 0.001 | 6.06 (2.93-12.54) | < 0.001 |

Statistical analyses were performed using logistic regression and results were reported as odds ratio (OR) and 95% confidence interval (95% CI). HBV, hepatitis B virus; HDV, hepatitis D virus.
