# Supplementary figures and images for: Demographics and outcomes of hepatitis B and D: A 10-year retrospective analysis in a Swiss tertiary referral center
Source: PLoS One. 2021 Apr 27;16(4):e0250347. doi: 10.1371/journal.pone.0250347 (PMC8078781; doi:10.1371/journal.pone.0250347)

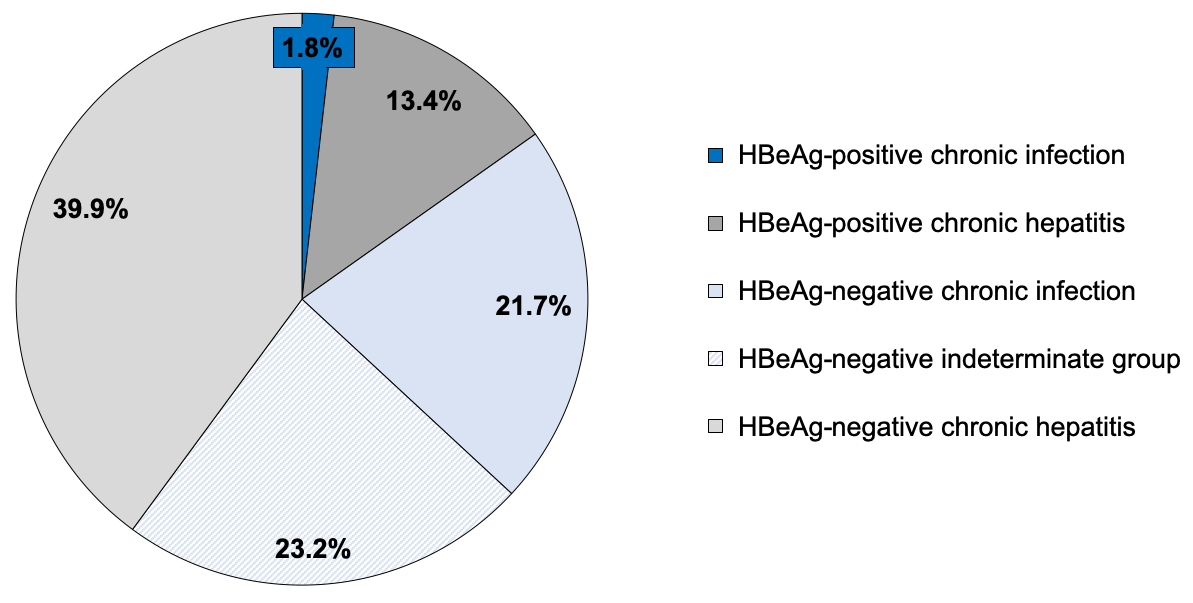

Supplement: S1 Fig — (TIF) [file pone.0250347.s002.tif]

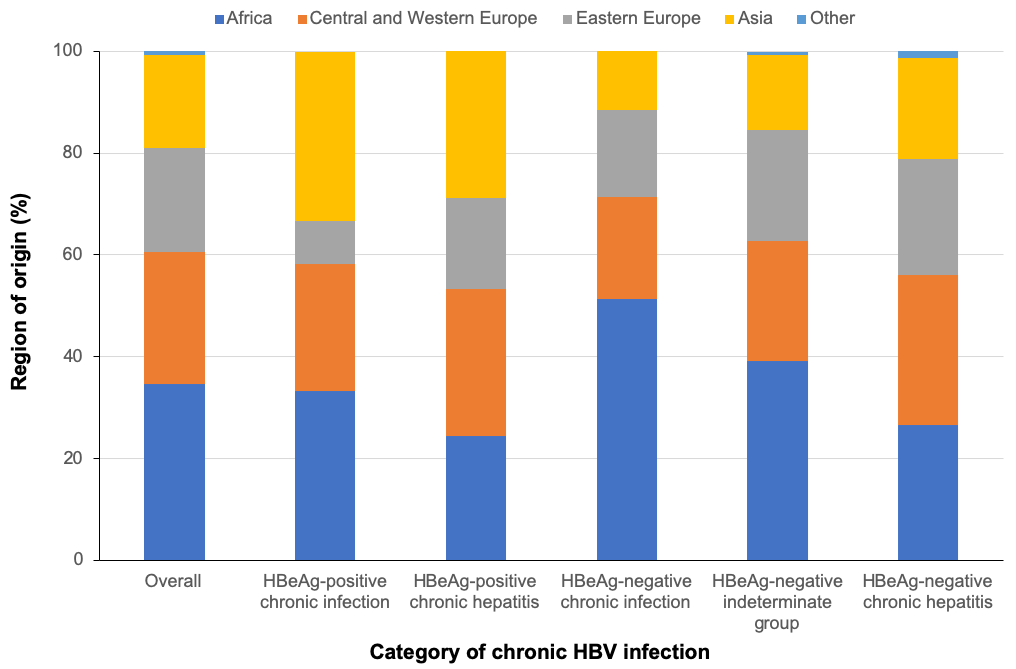

Supplement: S2 Fig — "Other" comprises patients from America and Australia. Results are expressed in percentages. HBV, hepatitis B virus. (TIF) [file pone.0250347.s003.tif]

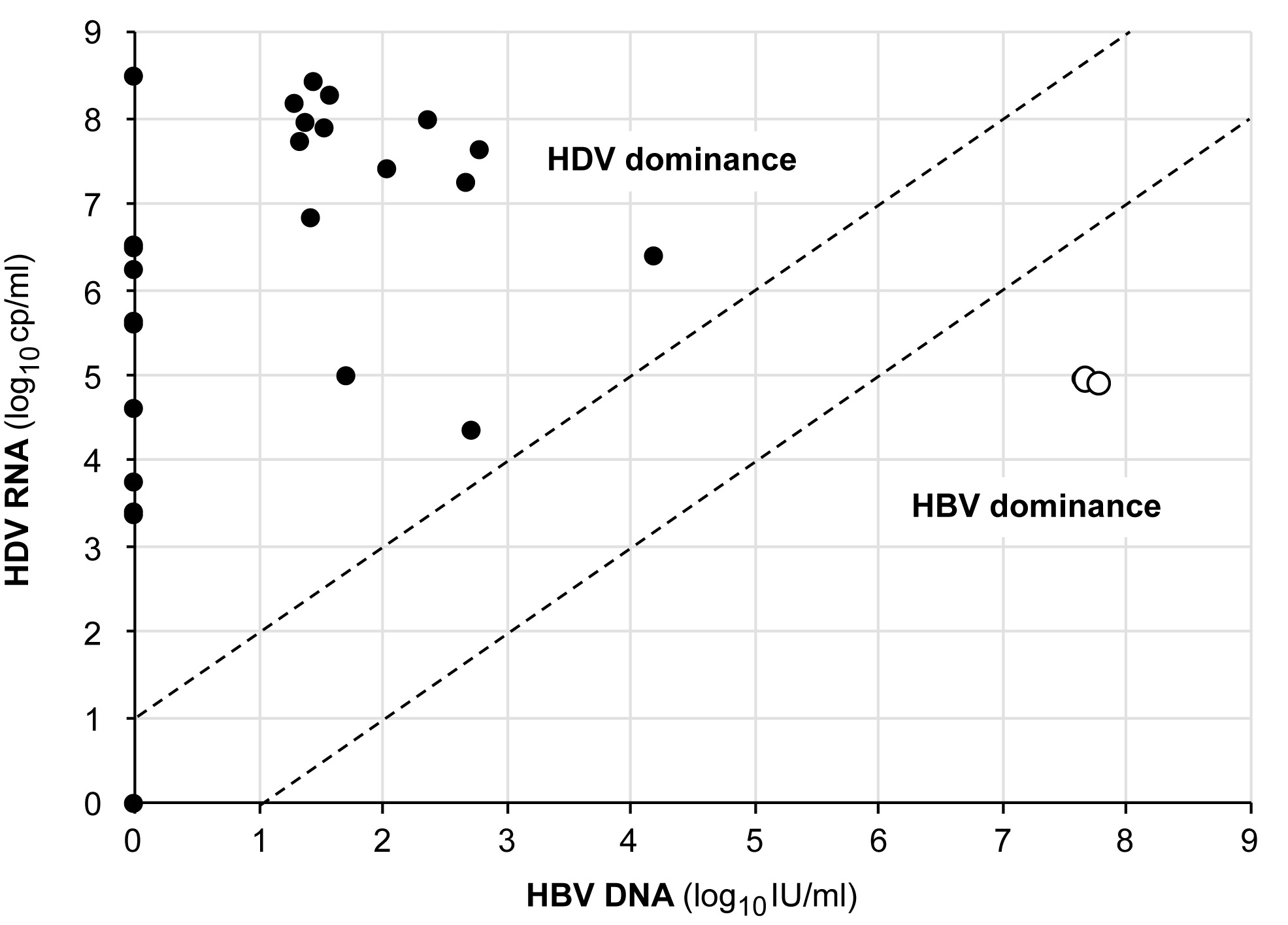

Supplement: S3 Fig — (TIF) [file pone.0250347.s004.tif]

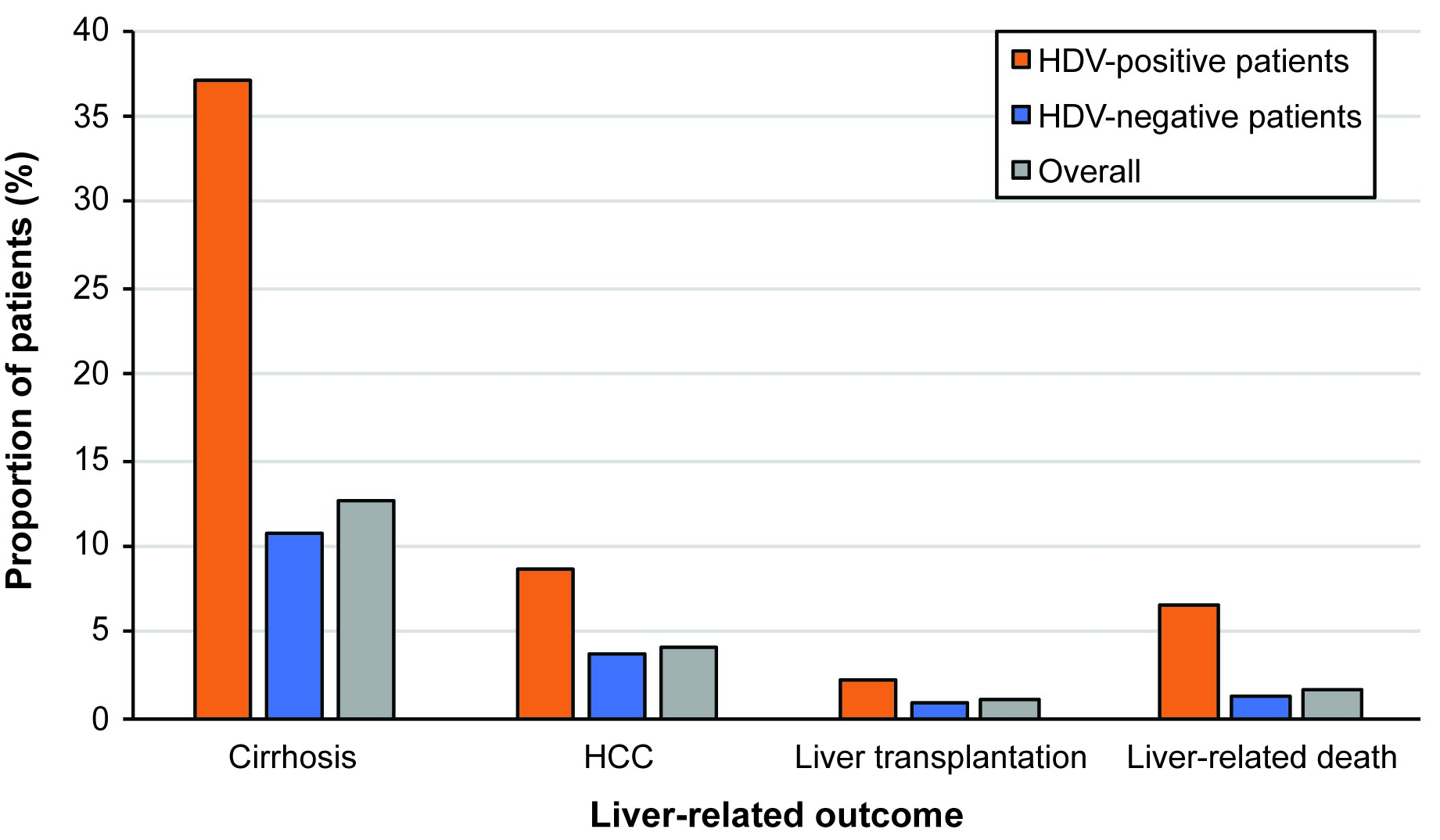

Supplement: S4 Fig — HCC, hepatocellular carcinoma. (TIF) [file pone.0250347.s005.tif]

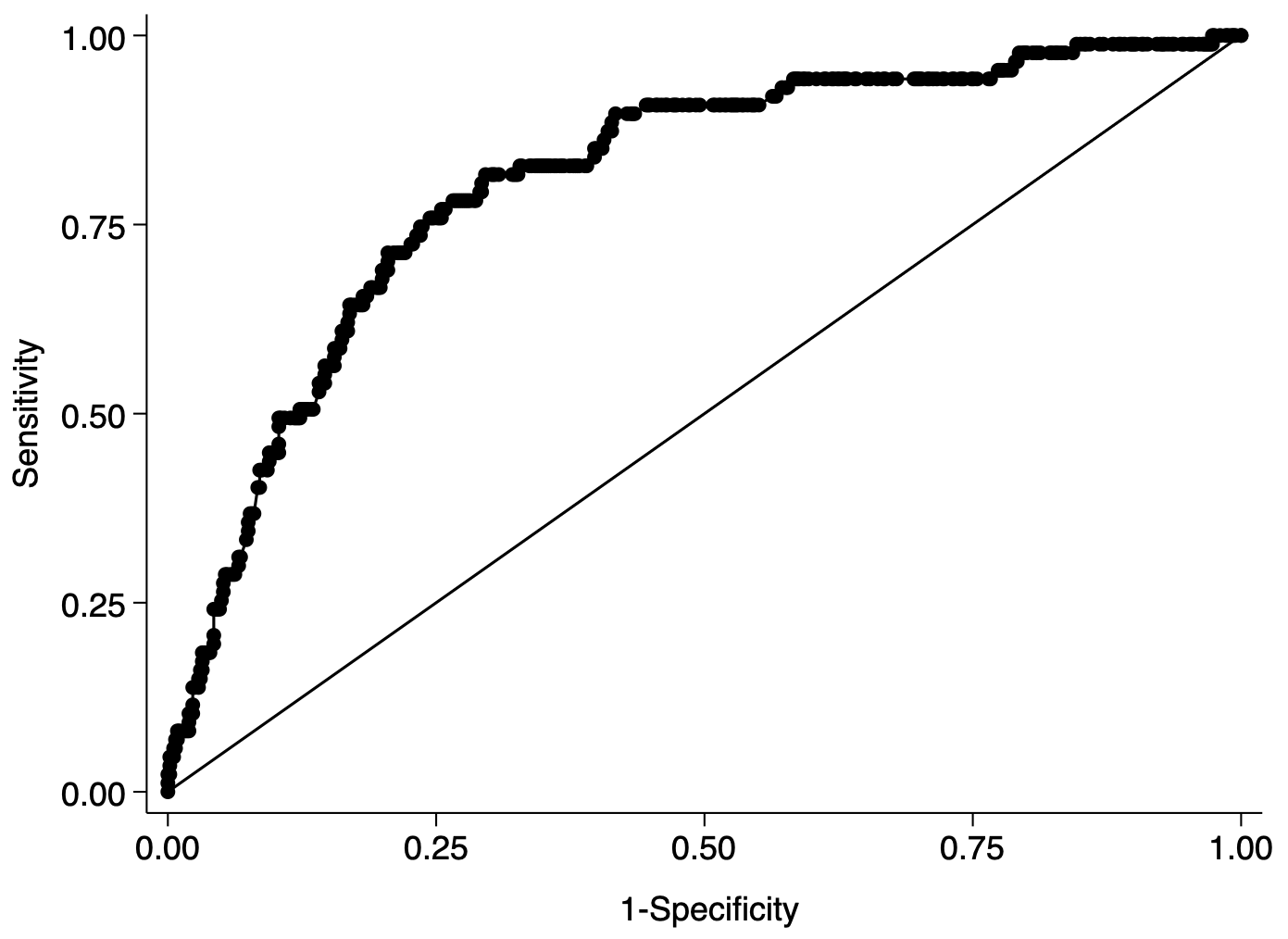

Supplement: S5 Fig — The area under the receiver operating characteristic curve for the multivariate model is 0.81. (TIF) [file pone.0250347.s006.tif]
